# Recurrent impetigo herpetiformis: case report

**DOI:** 10.11604/pamj.2017.27.219.12826

**Published:** 2017-07-24

**Authors:** Emmanuel Wekesa Wamalwa

**Affiliations:** 1 Faculty of Health Sciences, Egerton University, Njoro, Kenya

**Keywords:** Recurrent, impetigo herpetiformis, postpartum

## Abstract

Impetigo herpetiformis (pustular psoriasis of pregnancy) is a rare dermatosis of pregnancy that typically starts in the 2^nd^ half of pregnancy and resolves postpartum. It may recur in subsequent pregnancies. I present a case of 23 year old female gravida 4 para 3 with recurrent impetigo herpetiformis at 26 weeks gestation. She presented with a one month history of pustular lesions which responded to treatment with prednisone. She delivered at term with a favourable outcome. The disease resolved one month postpartum. This was the second recurrence of the disease. She had her first episode of impetigo herpetiformis during the second pregnancy. The disease recurred in the 3^rd^ pregnancy and resulted in a still birth.

## Introduction

Skin changes in pregnancy can be broadly divided into physiological, specific dermatoses of pregnancy, and other common skin diseases in pregnancy [1]. The specific dermatoses of pregnancy represent a heterogeneous group of severely pruritic inflammatory dermatoses associated exclusively with pregnancy or the immediate postpartum period [1]. Specific dermatoses of pregnancy are classified as: pemphigoid gestationis (herpes gestationis), pruritic urticarial papules and plaques of pregnancy, atopic eruption of pregnancy (eczema in pregnancy, prurigo of pregnancy, pruritic folliculitis of pregnancy) and pustular psoriasis of pregnancy (Impetigo herpetiformis).

Impetigo herpetiformis (pustular psoriasis of pregnancy) is a variant of pustular psoriasis, a specific dermatosis that occurs in pregnancy with the onset being in the 3rd trimester in majority of the cases. The condition was first reported by Ferdinand Ritter von Hebra in 1872. In his report, von Hebra [2] reported five cases of pregnant women with pustular lesions. All the cases had fetal death and four of the five women died.

Impetigo herpetiformis is an extremely rare condition. It is clinically and histologically similar to pustular psoriasis. There has been a debate on whether pustular psoriasis of pregnancy is a separate disease entity or a pustular stage of generalized pustular psoriasis occurring in pregnancy [3]. It is assumed by some authors to be a simple variant of generalized pustular psoriasis, representing a pustular stage of the disease, as a result of the hormonal changes of pregnancy or other factors that are not yet understood. However the authors have emphasized the need to consider this condition as a separate entity from generalized pustular psoriasis [4].

The underlying aetiology and specific pathogenesis of impetigo herpetiformis is largely unknown. The condition occurs in pregnancy in women who often have no history of pustular psoriasis. It is clinically manifested as erythema and pustular eruptions (usually without pruritus) and resolves postpartum. The disease is associated with placental insufficiency with sequelae such as miscarriage, fetal distress, fetal growth restriction, and stillbirths. Recurrence in subsequent pregnancies is common.

## Patient and observation

**Figure 1:**
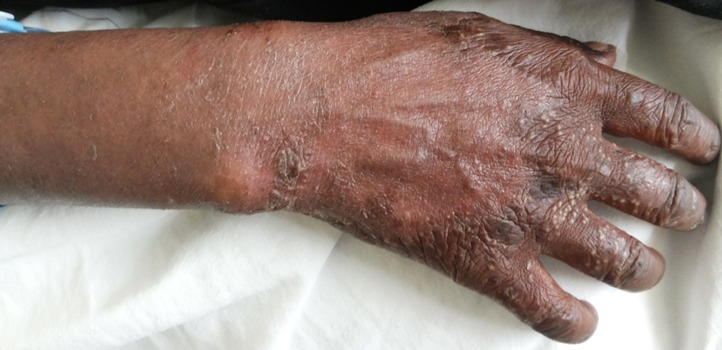
Hand lesions

**Figure 2:**
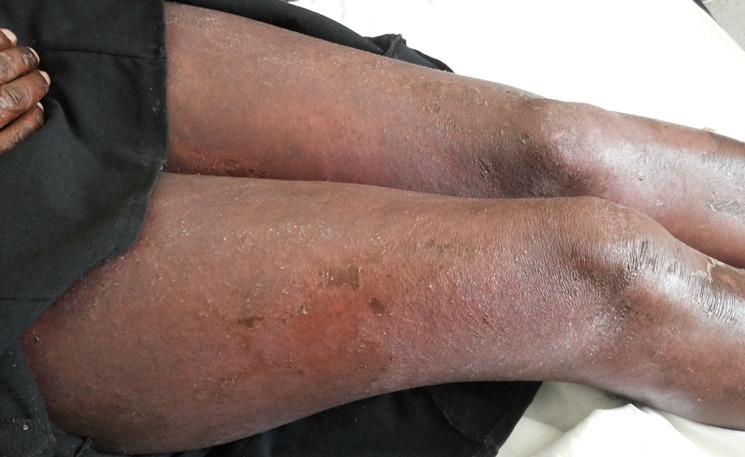
Resolving lower limb lesions

**Figure 3:**
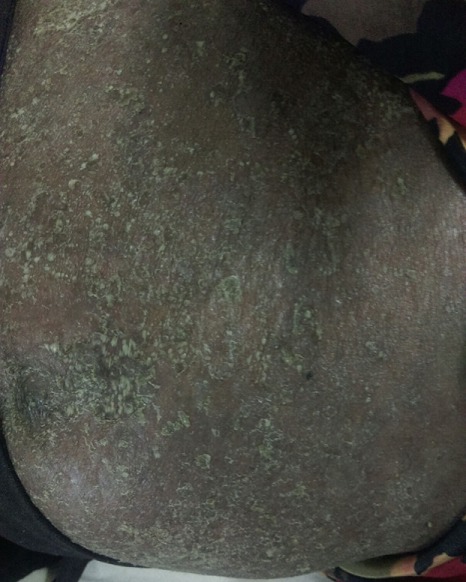
Abdominal lesions

I report a case of 23 year old African female, para 3 gravida 4. The patient presented to our facility (Moi Teaching and Referral Hospital, Kenya), on 23^rd^ January, 2016 at 26 weeks gestation. She presented with a one month history of a generalized body rash that was worsening. The rash started from the armpits and neck and later spread to the trunk and the rest of the body. She also reported history of fever and chills for five days. She had no personal and family history of psoriasis or any other dermatoses. She denied risk factors for pustular psoriasis such as smoking, and use of indomethacin and beta blockers. The patient was reviewed by the dermatology team and was found to have generalized pustular lesions as depicted in Figure 1, Figure 2, Figure 3. The body temperature was normal, 36.6°C.

The patient reported normal fetal movements. She had no history of per vaginal bleeding or drainage of liquor. On obstetric evaluation, she had a fundal height corresponding with gestational age. Obstetric ultrasound yielded normal findings. The patient was admitted for inpatient management. Laboratory investigations were conducted. Evaluation of blood slide for malaria parasite was done but found negative. A rapid HIV antibody test was done and she was found negative. A complete blood count was also done. The hemoglobin was 11.6 g/dl. White blood cell count was elevated at 13×10^9^/L (this may be normal in pregnancy) with neutrophilia of 81% (50-75). Platelet count normal, 414×109/L (330-400). Serum urea (2.2 mml/l), potassium (3.75mmol/l), sodium (133 mmol/l) and creatinine (30 μmol/l) were normal. Albumin level was low 30.3 g/l (35-55). Serum bilirubin and liver enzymes were essentially normal. Serum calcium levels, and parathyroid hormone levels were not evaluated. Histological studies were not performed.

The patient was admitted for five days. During admission, the patient was managed with prednisone 40mg/day, analgesics (oral paracetamol 1g thrice daily for 5 days) and prophylactic antibiotics (oral flucloxacillin 500mg four times daily for 5 days). Betason ointment 0.1% was applied on the lesion twice daily. The patient also received supportive management with intravenous fluids and psychosocial support during admission. She improved in five days and was discharged on prednisone 40mg/day for outpatient follow up. Upon improvement, prednisone dose was reduced to 20mg/day after three weeks, then to 15mg/day after another two weeks. The patient continued with prednisone 15mg/day until delivery.

She went into spontaneous labour and delivered vaginally at 38 weeks gestation. The outcome was a live female infant 3.0kg with APGAR score of 7 at 1 minute, 9 at five minutes and 10 at 10 minutes. There were no complications during labour and delivery. The lesions resolved one month postpartum. No further rebound or relapses were noted after resolution of the lesions. At the last review 10 months after delivery, the patient and her infant remained well without development of new skin lesions.

In her obstetric history, the patient had her first pregnancy in 2007. During this pregnancy, she did not develop any skin lesions. The pregnancy was uneventful and she delivered at term with a favourable outcome. In the second pregnancy (2009), she developed pustular skin lesions which started in the 3rd trimester near term and resolved two months after delivery. The details of her treatment for the lesions were not clear. She delivered at term with a favourable outcome. In 2013, she had her 3rd pregnancy and the skin lesions recurred. The lesions started at 22 weeks gestation. She did not receive any treatment. She had a still birth at term and labour was induced. She was not given any treatment and the lesions resolved one month postpartum.

### Ethical approval

I obtained written informed consent for patient information and images for publication. I also received approval from Moi University / Moi Teaching and Referral Hospital Institutional Research and Ethics Committee.

## Discussion

Impetigo herpetiformis is a dermatosis of pregnancy that clinically and histologically resembles generalized pustular psoriasis. It is a rare dermatosis with potential serious consequences for mother and the child. It tends to occur in the third trimester of pregnancy, although cases have been reported as early as the first trimester [5]. In this case, patient’s symptoms developed in the second trimester of pregnancy. Recurrence in subsequent pregnancies has been reported. In such cases, the disease tends to be more severe and occur at an earlier gestation, as was the case in our patient. Following resolution, flares have been reported postpartum, during menses and on use of oral contraceptives. The aetiology and pathogenesis of impetigo herpetiformis is not completely understood but may be related to hormonal changes in pregnancy, particularly progesterone. Hypocalcemia and hypoparathyroidism are considered to be aggravating factors.

Classically, impetigo herpetiformis is characterized by sterile pustules initially arising from intertriginous areas of the body, with subsequent involvement of the trunk and limbs. Some lesions may coalesce into large pus-filled bullae. The lesions are usually not pruritic as was the case in our patient. The patients may also have constitutional symptoms such as fever and generalized body malaise. The symptoms such as nausea, vomiting, dehydration, diarrhea, chills and convulsions have also been reported but these were absent in our patient [6,7].

Laboratory findings in impetigo herpetiformis include leukocytosis (with neutrophilia), negative bacterial culture of pustules, elevated ESR, hypocalcemia, hypoalbuminemia and hypophosphatemia [6]. Hypocalcemia may be related to hypoparathyroidism [8]. The reported patient had leukocytosis, neutrophilia, and hypoalbuminemia. Although the significance of histopathological examination of skin and placenta is not specified clearly, it may be useful in the diagnosis of this condition [9]. The histopathology of impetigo herpetiformis is similar to that of pustular psoriasis. The characteristic finding in an early lesion is the presence of collections of polymorphonuclear neutrophils in spongiotic foci in the epidermis, known as spongiform pustules of Kogoj [5].

Impetigo herpetiformis has been associated with poor pregnancy outcomes including miscarriages, premature rupture of membranes, still births and intrauterine growth restriction [10-12]. Our patient had a recurrence of impetigo herpetiformis in her 3^rd^ pregnancy which resulted in a still birth. The other two affected pregnancies had favourable outcomes. There are no standard guidelines for management of impetigo herpetiformis. Most cases have been successfully treated with prednisone which is considered the first line treatment. An initial dose of 15-30mg/day is usually sufficient, but the dose may be increased to 40-80mg/day in severe cases. Corticosteroids should be slowly tapered as the patient responds to treatment. Although our patient was treated successfully with prednisone, some unresponsive cases to prednisone have been reported [13-15]. Successful treatment with cyclosporine has been reported and this regime can be used as second line treatment [6,16,17]. Antibiotics may be used to prevent and treat infections. It is reported that taking parenteral calcium, vitamin D, infliximab and pyridoxine in high doses, as well as chorionic gonadotropin, is effective for impetigo herpetiformis during pregnancy [9]. Taking methotrexate, retinoids (such as acitretin) and ultraviolet A (PUVA) may be helpful for treatment of impetigo herpetiformis after delivery [12, 18-20].

## Conclusion

The case had classical features of impetigo herpetiforms. The symptoms of this condition start in pregnancy and resolve postpartum, with risk of recurrence in subsequent pregnancies. The affected pregnancies may have bad outcomes such as stillbirths. Majority of patients respond to corticosteroid treatment and this should be used as first line in patients with impetigo herpetiformis. The affected patients should be followed up in subsequent pregnancies to observe for recurrence.

## Competing interests

The authors declare no competing interest.
